# Iodine-125 plesiotherapy for murine tumor treatment

**DOI:** 10.1186/s13014-025-02657-0

**Published:** 2025-05-16

**Authors:** Audrey Glory, Rodin Chermat, Julie Lafontaine, Danh Tran-Thanh, Philip Wong

**Affiliations:** 1 Centre de recherche du CHUM, Montréal, Québec Canada; 2 µFO Lab, Institute of Biomedical Engineering, Polytechnique Montréal, Montréal, Québec Canada; 3 Département de Pathologie, Université de Montréal, Montréal, Québec Canada; 4 Department of Radiation Oncology, University of Toronto, Toronto, ON Canada; 5 Radiation Medicine Program, Princess Margaret Cancer Centre, University Health Network, Toronto, Canada

**Keywords:** Plesiotherapy, Radiotherapy, Iodine-125, In vivo tumor treatment, Cost-effectiveness

## Abstract

**Background:**

Radiotherapy (RT) is one of the four pillars of cancer treatment. Plesiotherapy, or contact brachytherapy, involves irradiating a tumor by placing small radioactive sources directly on the skin’s surface above the tumor. In this study, we evaluated the efficacy of a novel local external radiation technique using iodine-125 seeds enclosed within a 3D-printed case, positioned externally on the tumor surface.

**Methods:**

First, the protocol was tested on the skin of NodRag1 mice with doses up to 10 Gy (at the skin), and the results demonstrated no signs of skin toxicity. Subsequently, this protocol was used to locally irradiate subcutaneous MDA-MB-231 triple-negative breast cancer and MCA-205 fibrosarcoma tumors via a single 10 Gy dose at the tumor center.

**Results:**

RT significantly hindered tumor growth, with irradiated tumors being approximately half the size of nonirradiated tumors on the same day. Importantly, the irradiated mice exhibited no apparent systemic side effects, as evidenced by stable body weight and unaffected behavior, including alertness, appearance, and activity levels. Moreover, no instances of skin toxicity were observed.

**Conclusions:**

This in vivo plesiotherapy protocol offers a straightforward and cost-effective means of advancing research on RT in a variety of laboratory settings.

**Supplementary Information:**

The online version contains supplementary material available at 10.1186/s13014-025-02657-0.

## Background

Radiotherapy (RT) is one of the four main strategies used in cancer treatment, along with surgery, chemotherapy, and immunotherapy [1]. Used in approximately half of cancer patients, either on its own or in combination with other modalities, RT can be delivered via external beam RT (EBRT) or brachytherapy (BT) [2]. In EBRT, the patient is placed under a linear accelerator (LINAC) that emits X-rays at prespecified energies and shapes to irradiate the tumor from the outside inward. Conversely, in BT, small radioactive sources or devices are placed inside or near the tumor to irradiate it from the inside out. Both approaches exist in a large array of setups, dose rates and radiation types and thus generate a variety of biological effects and efficacy ranges [2]. Plesiotherapy, also known as contact BT, is the irradiation of a tumor using small radioactive sources or devices that are placed on the surface of the skin with the tumor underneath [3]. According to the National Comprehensive Cancer Network (NCCN) guidelines, RT is recommended as a treatment option for most patients with breast cancer and soft tissue sarcoma (STS) in conjunction with conservative surgery [4]. Breast cancer is the most prevalent cancer in the world. In the U.S., its incidence has risen in most of the past four decades, increasing by 0.5% annually in the last decade [5]. BT is recommended for breast cancer, with low-dose-rate (LDR) BT having been shown to be efficient for partial breast irradiation or as a boost after EBRT [6]. However, high-dose rate (HDR) brachytherapy is often preferred for accelerated partial breast irradiation, a more convenient outpatient procedure [7]. STSs are highly heterogeneous tumors that can arise in any connective or other nonepithelial tissue. They represent only 1% of all adult malignancies and 7% of all cancers in children up to 15 years of age [8, 9]. Again, BT is an essential component of STSs treatment, with clear evidence of improved local control following surgery. LDR BT is recommended pre-operatively, post-operatively, or in combination with EBRT either as a boost or as the main irradiation modality [10]. LDR BT for STS is described as convenient, effective and spares normal tissue, making it ideal for small high-grade disease or re-irradiation, especially in the context of pediatric patients [6]. To improve the treatment options for breast cancer and STS patients, among others, there is a need to facilitate the generation of preclinical data on the use of RT combined with various novel molecular agents, such as modulators of the immune system.

For the study of tumor irradiation in vivo, animals can be entirely irradiated (using Gamma or X-rays) [11, 12]; the tumor can be locally irradiated, for example, by using a clinical linear accelerator or another EBRT system [13–15]; or by the insertion or application of radioactive BT seeds [16, 17]. In the case of global irradiation, all fast-dividing cells will be impacted, leading to the depletion of the immune system and the need to graft new immune cells to ensure the survival of the animal [11, 12]. Although clinical machines provide targeted irradiation, they are not readily available and, if so, are generally dedicated to clinical use [18]. Small irradiators, also known as “cabinet irradiators”, have been specifically designed for the irradiation of in vitro samples and small animals. However, their high cost and footprint coupled with the need for cumbersome setups to ensure targeted irradiation in animals make them ill-suited for the democratization of in vivo radiotherapy research. On the other hand, local irradiation of tumors by direct intratumoral implantation of BT seeds is inconvenient due to the small size of the tumors in animal models, such as mice, but the use of BT seeds themselves presents experimental and practical advantages compared to previously listed alternatives. As such, plesiotherapy is a simple, low-cost and low-footprint methodology for animal irradiation. As past in vivo plesiotherapy techniques require the use of a handheld device [19–21], it is difficult to reproduce these procedures in a large number of animals. Therefore, in the present study, we assessed the efficacy of a new local external technique for irradiating tumors in vivo using iodine-125 (I-125) seeds placed externally in a 3D-printed case on the surface of the tumor.

## Methods

### Irradiation setup

**Fig. 1:**
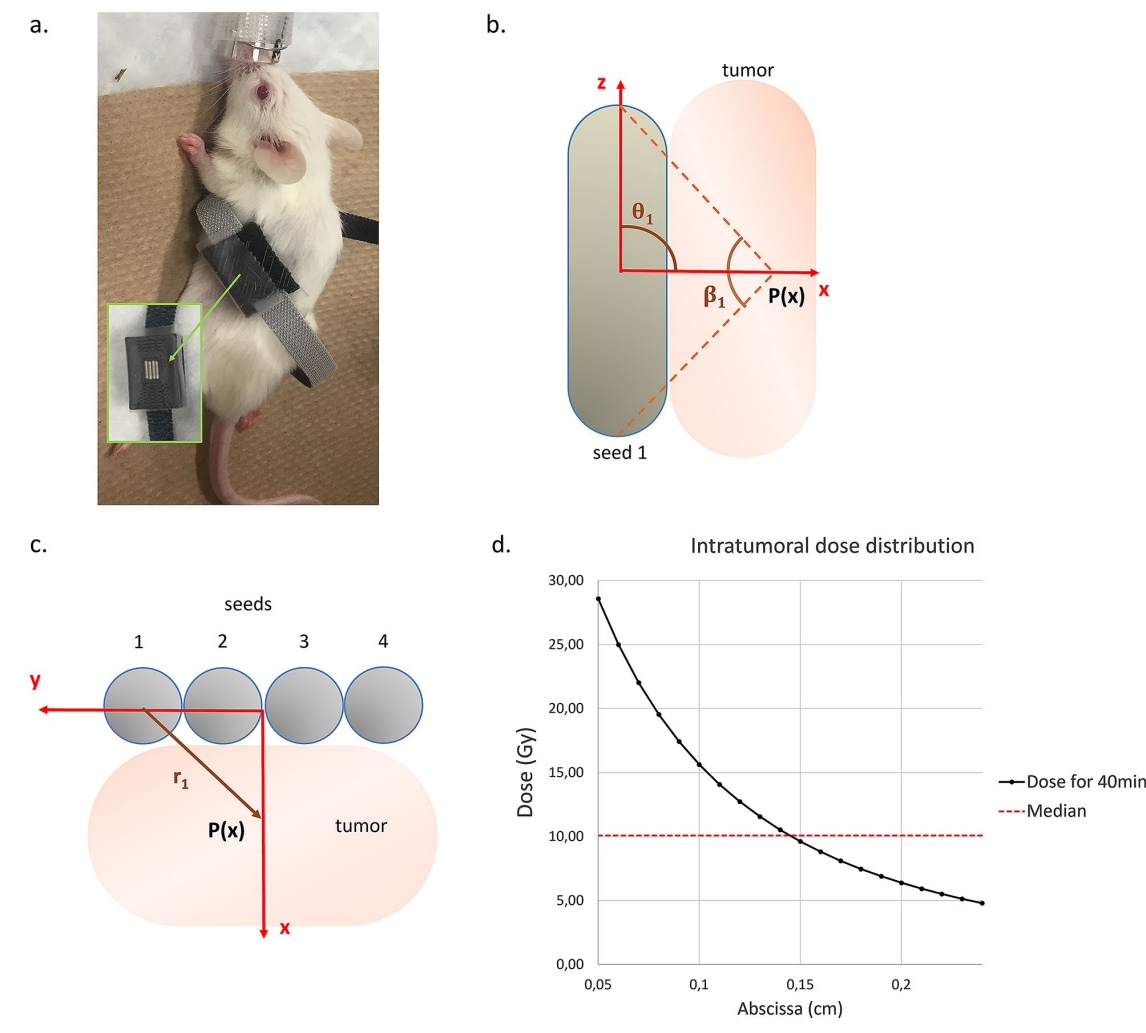
Dose calculation. **a**. Representative image of the local irradiation of mice using iodine 125 seeds encased in a 3D-printed plastic bracelet and placed directly above the tumor. **b**. Side view schematic of the setup geometry. Seed 1 is taken as an example to visualize the geometric variables $\:\theta\:$_i_ and $\:\beta\:i$. **c**. Top view schematic of the setup geometry. Seed 1 is taken as an example to visualize the geometric variable r_i_. **d**. TG-43-based estimation of the intratumoral dose distribution across the central axis of the tumor using the 2D-line source approximation. A median value of 10.07 Gy is indicated by a dotted red line

Four I-125 seeds (IsoAid, Port Richey, FL, USA) with an initial activity of 12 mCi were manually inserted into a custom-made 3D-printed polylactic acid (PLA) case mounted on a flexible Velcro^®^ bracelet and secured using transparent tape. Briefly, the case is a 15 mm large x 12 mm long x 3 mm thick block with a 4.5 mm large x 3.5 mm long x 1 mm deep socket located in its center (please refer to the stl file in Supplementary material). These dimensions were specifically selected to ensure easy insertion, tight hold and subsequent removal of four I-125 seeds within the socket, in perfect contact with one another. The seeds were then placed in direct contact with the skin on top of the subcutaneous tumor if present (Fig. 1a).

### Dose calculation

The intratumoral dose distribution was calculated with our 4-seed irradiation setup using the previously described TG-43 2D-line source formalism [22]. Briefly, the dose rate from a seed “i” at a point P(r_i_; θ_i_) is defined by:

1 $$\:\dot{D}\left({r}_{i},\theta\:\text{i}\right)=\:S\text{k}*\varLambda\:*\frac{GL(ri,\theta\:\text{i})}{GL(1,\pi\:/2)}*g\text{L}\left(r\text{i}\right)*F(r\text{i},\theta\:\text{i})$$

where r_i_ is the radial distance from the center of the seed (cm), θ_𝑖_ is the polar angle (rad), S_k_ is the air kerma strength (cGy*cm^2^/h), Λ is the dose rate constant (cm^−2^), GL(r_i_; θ_i_) is the 2D geometric function (cm^−2^), g_L_(r_i_) is the radial dose function and F(r_i_; θ_i_) is the 2D anisotropy function.

Under the 2D-line source approximation:

2 $$\:G\text{L}(r\text{i};\:\theta\:\text{i})=\:\frac{\beta\:i}{L*ri*\text{s}\text{i}\text{n}\left(\theta\:i\right)}$$

where L is the active length of the seed and 𝛽_𝑖_ is the opening angle (rad).

The seed dimensions, tabulated values and fitting parameters for the various functions were taken from the CLRPv2 database [22].

Given our irradiation setup, we defined the x-axis such that ∀_i_, θ_i_ = π/2 (Fig. 1b). Therefore, F(r_i_,θ_i_) = 1 for any P(x), and (a) is simplified to:

3 $$\:\dot{D}\left({r}_{i},\pi\:/2\right)=\:S\text{k}*\varLambda\:*\frac{GL(ri,\pi\:/2)}{GL(1,\pi\:/2)}*g\text{L}\left(r\text{i}\right)$$

Considering that the seeds are perfectly aligned with no spacing and that they are in perfect contact with a 2 mm thick tumor, the total intratumoral dose distribution was then calculated by summing the contribution of each seed across the x-axis (Fig. 1c):

4 $$\:\dot{D}\:\left(x\right)={\sum\:}_{i=1}^{4}\dot{D}\:\left({r}_{i}\right)$$

With this approach, our intratumoral median value target of 10 Gy was met with an irradiation time of 40 min (10.07 Gy; Fig. 1d).

### In vivo studies– skin toxicity

Eight NodRag1 female mice (from a local colony) were divided into three treatment groups according to Table 1. All the mice were shaved on both flanks, and their skin was graded prior to irradiation according to Table 2, as described by Iwakawa et al. [23]. The mice were anesthetized with isoflurane at 1.5-2% with 0.5 L/min O_2_ and locally irradiated on their flanks using I-125 radioactive seeds. The contact time between the seeds and the skin was calculated so that the skin would receive a radiation dose of approximately 4, 8–10 Gy (target median dose). All the mice were monitored daily for 30 days for skin toxicity, as shown in Table 2. Skin toxicity is considered mild between the scores of [-1] and [1.5] and it is considered severed at the score of [1.5] and above. Pictures of the irradiated area were taken once per week. Mice were weighed prior to irradiation and after 6 days. All protocols were approved by the CR-CHUM Institutional Animal Care Committee and were conducted in accordance with the Canadian Council on Animal Care (CCAC) guidelines and ARRIVE guidelines. The number of animals used was kept low to comply with the three Rs (Replacement, Reduction, Refinement) policy of the CCAC. We adopted a 3 + 3 design, common in clinical trials, that consists of testing a small number of individuals first and adding 3 more individuals if one of them presents signs of toxicity. Given that this was not the case here, no animals were added to the skin toxicity study.

**Table 1:** Distribution of mice in the three treatment groups

Treatment	Number of mice
Left flank − 0 Gy	3
Right flank − 4 Gy	
Left flank − 4 Gy	3
Right flank − 8 Gy	
Left flank − 0 Gy	2
Right flank − 10 Gy	
**Total number of mice**	8

**Table 2:** Mouse skin reaction grading tool (from Iwakawa et al.)

Score	Observation
0.5	50/50 doubtful if there is any difference from normal
1-	Definite but slight abnormality
1	Definite abnormality with reddening
1+	Severe reddening and/or white scales and/or puffiness
1.5	Moist breakdown in one very small area, with scaly or crusty appearance
1.5+	Moist desquamation in small areas (more definite than 1.5)
2	Breakdown of large area, possibly moist in places
2.5	Breakdown of large areas of skin with definite moist exudate
3	Breakdown of moist skin with moist exudate
3.5	Complete moist breakdown of limb - often stuck to body

### In vivo studies– tumor growth

MDA-MB-231 human triple negative breast cancer (TNBC) cells (a generous gift from Dr. Mes-Masson’s laboratory at the CR-CHUM, authenticated via STR typing) were cultured as monolayers in Dulbecco’s modified Eagle’s medium (DMEM) supplemented with 10% fetal bovine serum (FBS) and 1% penicillin streptomycin. The cells were maintained at 37 °C in a humidified atmosphere of 5% CO_2_. NodRag1 female mice (from a local colony) were subcutaneously injected with 1.10^6^ MDA-MB-231 cells in the left flank. Twelve days after injection, the mice were randomly divided into two groups: nontreated (control, *n* = 7) and irradiated (*n* = 9). The average size of the tumors on day 12 was 23.76 mm^3^.

MCA-205 murine fibrosarcoma cells (a generous gift from Dr. Routy’s laboratory at the CR-CHUM, authenticated via STR typing) were cultured in monolayers in Roswell Park Memorial Institute (RPMI) 1640 medium supplemented with 10% FBS, 2 mM L-glutamine and 1% penicillin streptomycin at 37 °C in a humidified atmosphere of 5% CO_2_. C57Bl/6 female mice (from Charles River Laboratories, Saint-Constant, QC, Canada) were subcutaneously injected with 5.10^5^ MCA-205 cells in the left flank. Ten days after injection, the mice were randomly divided into two groups: nontreated (control, *n* = 6) and irradiated (*n* = 9). The average size of the tumors on day 10 was 26.53 mm^3^. One mouse from the MCA-205 control group was excluded because its tumor did not grow.

The mice from the irradiated group were anesthetized with isoflurane at 1.5-2% with 0.5 L/min O_2_ and locally irradiated on the surface of their tumors using I-125 radioactive seeds. The contact time between the seeds and the tumors was calculated so that the irradiated tumors would receive a radiation dose of approximately 10 Gy (target median dose at the center of the tumor). This calculation was based on the mean height of the tumors. The mice from the control group were anesthetized using the same conditions and for the same amount of time as the irradiated mice. Tumor volume was measured every 2–3 days using a caliper according to the following formula: (π/6)*(height*width*length). MDA-MB-231 tumor-bearing mice were sacrificed on day 31 after injection. MCA-205 tumor-bearing mice were sacrificed on day 31 after injection, or when the tumor diameter reached 2 cm. All the mice were weighed once a week and were monitored daily for local side effects, alertness and appearance. The toxicity to the skin was graded according to Table 2. All protocols were approved by the CR-CHUM Institutional Animal Care Committee and were conducted in accordance with the Canadian Council on Animal Care (CCAC) guidelines and ARRIVE guidelines.

### Statistics and analysis

The data are presented as the means +/- standard errors of the means. Given that a Gaussian distribution cannot be assumed, a mixed-effects model with Dunnett’s multiple comparisons test or Wilcoxon matched-pairs signed rank test was used to analyze the data. The software used was GraphPad Prism 10 (GraphPad Software, Inc., San Diego, CA, USA). *p* < 0.05 was considered to indicate statistical significance.

To analyze the impact of irradiation on tumors in the tumor growth in vivo study (Fig. 4), the tumor volume for each mouse was normalized by its tumor volume on the day of irradiation (or anesthesia for mice in the control group). Then the mean tumor volume for the control and irradiated groups was calculated to compare between the two groups.

## Results

### Local external irradiation using I-125 is safe for mice

**Fig. 2:**
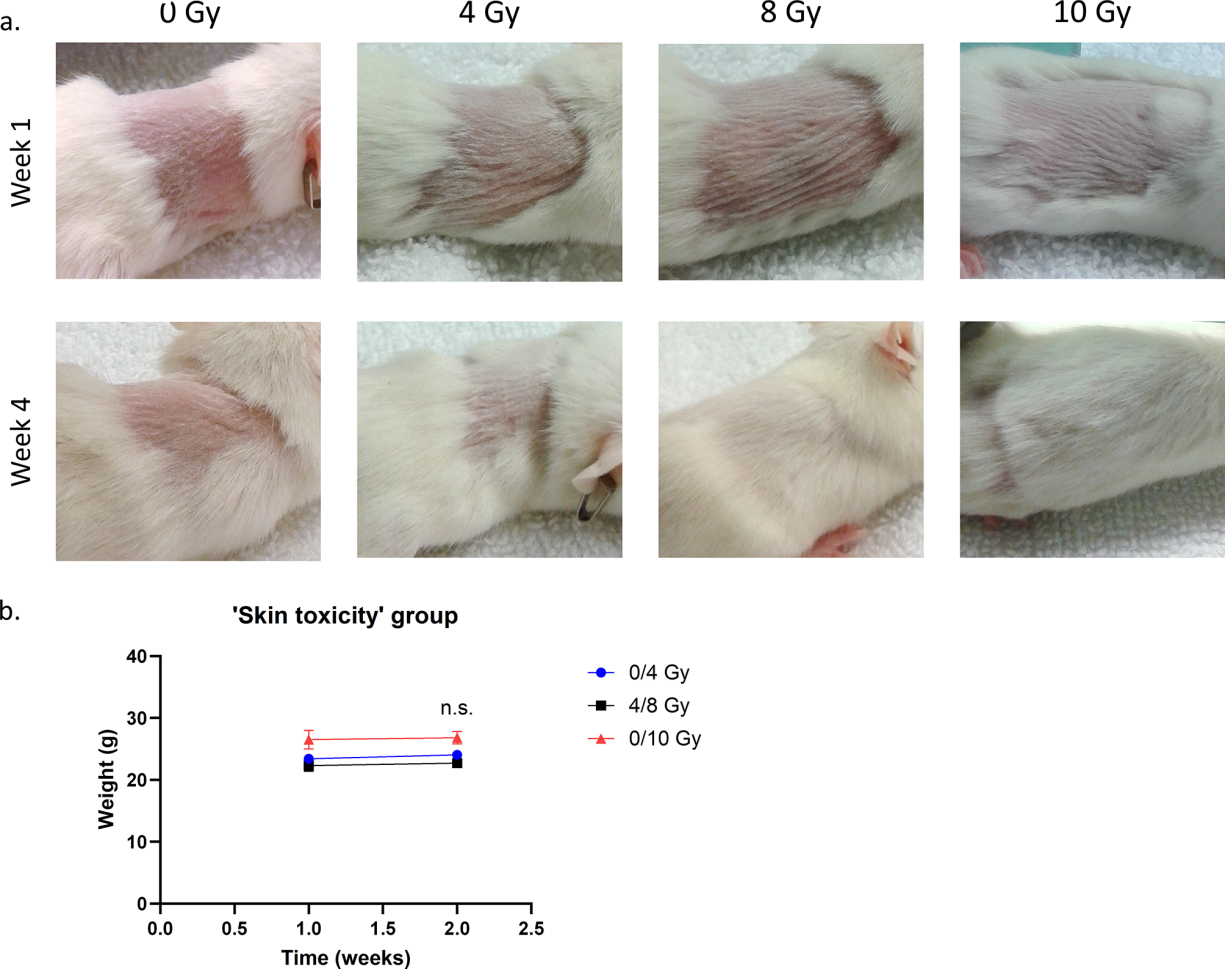
In vivo skin toxicity experiment **a**. Representative images of the skin of irradiated (4, 8 and 10 Gy) and nonirradiated (0 Gy) mice before treatment (week 1) and at the end of the study (week 4). **b**. Body weights of the mice used in the ‘skin toxicity’ in vivo experiment. Mice were weighed at weeks 1 and 2. The irradiation took place on week 1, immediately after weighing. The curves represent the mean weights of the mice in the 0/4 Gy group (blue), the 4/8 Gy group (black) and the 0/10 Gy group (red). Error bars represent the standard error of the mean (Wilcoxon matched-pairs signed rank test)

To validate our irradiation protocol, the first step was to confirm the absence of skin toxicity, as defined in Table 2. There was no sign of skin toxicity (0/3.5 on the Table 2 grading tool) in any of the mice, independent of the irradiation dose (from 0 Gy to 10 Gy at the skin) (Fig. 2a). The pictures presented in Fig. 2a are representative of the appearance of the mice’s skins during the experiment, as a grading of 0/3.5 was maintained throughout. The weights of the mice were stable before (week 1) and after (week 2) irradiation (Fig. 2b).

**Fig. 3:**
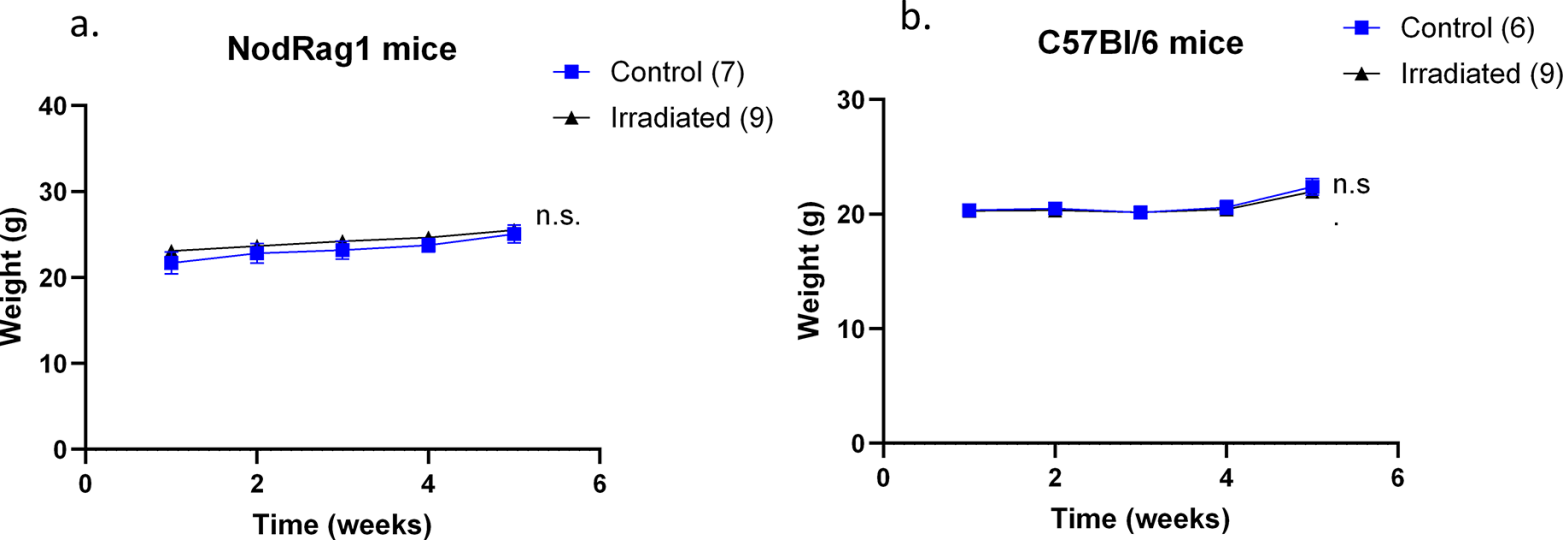
Body weights of the mice used in the ‘tumor growth’ in vivo experiments. NodRag1 mice (**a**) were injected with MDA-MB-231 cells, and C57Bl/6 mice (**b**) were injected with MCA-205 cells. Mice were weighed once a week for the duration of the experiments. The irradiation took place during week 2. Each curve represents the mean weight of mice in the control group (blue) or the irradiated group (black). Error bars represent the standard error of the mean (Wilcoxon matched-pairs signed rank test)

It was therefore safe to validate our irradiation protocol in tumor-bearing mice. The weights of the NodRag1 mice in the control and irradiated groups were similar (Fig. 3a), as were those of the C57Bl/6 mice (Fig. 3b). It is estimated that just under the seeds, the skin of the mice received a dose of approximately 28.6 Gy. However, there were no local complications (0/3.5 on the Table 2 grading tool; i.e., no acute radiation-induced dermatitis or other skin-related problems). There was no difference between control and irradiated mice in terms of behavior or signs of pain (data not shown).

### Local external irradiation of tumors with I-125 slows tumor growth

**Fig. 4:**
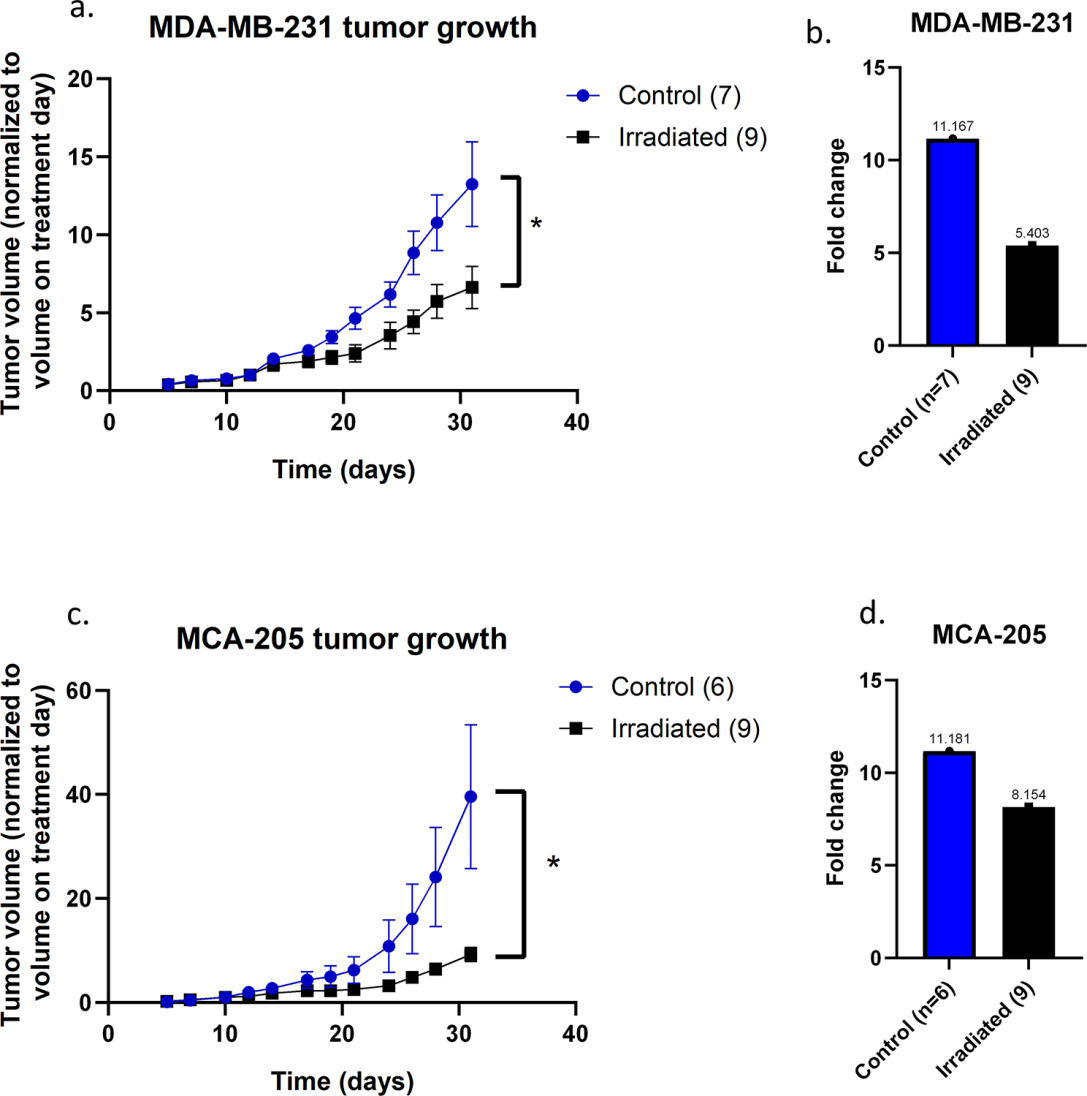
In vivo evaluation of the irradiation of solid tumors using I125 seeds. **a**. Mean tumor volume of MDA-MB-231 tumors; each tumor volume was normalized to the same tumor volume on treatment day (day 12): nontreated tumors (control, blue, *n* = 7) and irradiated tumors (irradiated, black, *n* = 9). Cells were injected into NodRag1 mice on day 0. **b**. Fold change between the mean tumor volume on the day of irradiation and the mean tumor volume on the last day of the experiment. **c**. Mean tumor volume of MCA-205 tumors. Each tumor volume was normalized to the same tumor volume on treatment day (day 10): nontreated tumors (control, blue, *n* = 6) and irradiated tumors (irradiated, black, *n* = 9). C57Bl/6 mice were injected on day 0. **d**. Fold change between the mean tumor volume on the day of irradiation and the mean tumor volume on the last day of the experiment. For **a** and **c**, error bars represent the standard error of the mean (mixed-effects model with Geisser–Greenhouse correction). In c, please note that the error bars for the irradiated group are included and are smaller than the squared symbol

Irradiation of the tumors at 10 Gy (target median dose) using this protocol resulted in a significant delay in growth for irradiated tumors compared to control tumors for both MDA-MB-231 TNBC tumors (Fig. 4a) and MCA-205 sarcoma tumors (Fig. 4c). For the MDA-MB-231 tumors, the relative mean volume two days after irradiation (day 14) was 2.06 for the control group and 1.71 for the irradiated group, indicating a difference of 1.21-fold. Nine days after irradiation (day 21), the control tumors were 4.64 times larger, while the treated tumors were 2.4 times larger. At this point, the tumor growth delay was 5.33 days (i.e., irradiated tumors required 5.33 days longer to reach the same size as control tumors). Nineteen days after irradiation (day 31, the last day of the experiment), the relative mean volume was 13.24 for the control group and 6.63 for the irradiated group. The tumors in the control group were twice as large as those in the irradiated group (Fig. 4a). The fold change between the relative mean tumor volume on the day of irradiation and the relative mean tumor volume on the last day of the experiment was 11.17 for control tumors and 5.4 for irradiated tumors, indicating a 51.62% decrease (Fig. 4b). All 16 mice survived and were monitored for 31 days, which marked the end of the experiment. For the MCA-205 tumors, the relative mean volume two days after irradiation (day 12) was 1.97 for the control group and 1.19 for the irradiated group, indicating a difference of 1.66-fold. Eleven days after irradiation (day 21), the relative mean volume was 6.21 for the control group and 2.53 for the irradiated group, with tumors in the control group being 2.46 times larger than those in the irradiated group. At this point, the tumor growth delay was 6.75 days (i.e., irradiated tumors required 6.75 days longer to reach the same size as control tumors). Twenty-one days after irradiation (day 31, the last day of the experiment), the relative mean volume was 39.6 for the control group, compared to 9.21 for the irradiated group, with tumors in the control group being 4.3 times larger than those in the irradiated group (Fig. 4c). The fold change between the relative mean tumor volume on the day of irradiation and the relative mean tumor volume on the last day of the experiment was 11.18 for control tumors and 8.15 for irradiated tumors, representing a 27.07% difference (Fig. 4d). Two out of six mice in the control group and three out of nine mice in the irradiated group were euthanized between day 24 and the end of the experiment due to tumor ulceration. Completely removing those 5 mice from the graph in Fig. 4c does not change its conclusion (data not shown).

Upon necropsy, the tumors were harvested, fixed, paraffin-embedded, sliced and stained using hematoxylin and eosin (H&E). Unfortunately, at that time (18 or 20 days after irradiation, for MDA-MB-231 and MCA-205 tumors respectively) it was not possible to witness the cellular damages caused by a single irradiation with I-125. Our findings show that most tumors (irradiated or not) exhibited a necrotic core (between 10% and 40% of the tumor). In approximately 25% of tumors, this necrotic zone extended toward the top part of the tumor (closer to the skin), but this was true for both irradiated and non-irradiated tumors (data not shown).

The skin on top of the tumors was also harvested, but the mice used in the study were also part of a larger protocol that required a surgical opening of the skin on top of the tumor 48 h after irradiation. As such, it was very difficult to differentiate damage that would result from irradiation and from surgery (data not shown). Additionally, MCA-205 tumors tend to create ulcers on the skin directly on top of the tumor, further complicating the analysis of the skin.

## Discussion

To address the need for simple, low-cost, low-footprint and accessible RT, we developed a protocol for the external irradiation of tumors using I-125 seeds. First, the protocol was tested on the skin of NodRag1 mice at various radiation doses up to 10 Gy (at the skin), without exhibiting any skin toxicity. This protocol was subsequently used to locally irradiate MDA-MB-231 TNBC and MCA-205 fibrosarcoma subcutaneous tumors via a single dose of 10 Gy (at the center of the tumor). The application of RT was highly effective at impeding tumor growth. Notably, irradiated tumors exhibited a significant reduction in size, reaching dimensions as small as half that of nonirradiated tumors on the same day. The irradiated mice showed no discernible global side effects, as evidenced by stable body weight and the absence of alterations in behavior, including alertness, appearance, and activity levels. Furthermore, there was no observed skin toxicity, confirming our previous observations. The single dose of 10 Gy (target median dose) used in this study has proven to be very effective at decreasing tumor growth and could be reduced to better determine the effect of combinations with other modalities. This could be achieved through simple dose reduction, which is easily achievable by decreasing the irradiation time. The main limitation of this study is the lack of histopathological evidence to showcase the dose distribution inside the tumor and document the lack of toxicity on the skin. The protocol was designed to be able to monitor changes in tumor growth, therefore the mice were kept alive for too long to be able to see the potential impacts of the I-125 irradiation on tumors at the time of necropsy. To address this limitation, future protocols should include mice sacrificed 48 h and 96 h after irradiation with I-125 seeds, to harvest tumors and skin samples. These samples could then be stained with H&E to observe irradiation-induced damage. Other markers such as Ki67 (proliferation), CD31 (angiogenesis), or TUNEL staining (apoptosis) could also be used to better document the dose distribution from I-125 irradiation.

Currently, there are three possible means of administering RT to small animals for preclinical studies. First, complete irradiation leads to health complications, as all organs receive the same dose of RT [11, 12]. Second, local irradiation of the tumor using either human EBRT setups or smaller machines is more adaptable to in vivo tests [13–15]. In both cases, this requires the research team to have access to a hospital with an RT facility or to purchase a dedicated cabinet irradiator adapted for small mammals for more than a hundred thousand USD. The cost and complexity of access and use drastically limits animal throughput and hinders the ability of small research groups to conduct in vivo radiotherapy research [18]. The third option is BT, i.e., the use of radioactive seeds to locally irradiate a tumor. BT can be conducted by inserting a radioactive seed into the tumor, which requires proper tools and skills, as tumors in mice can be quite small [24–26]. In addition, a minimal tumor size must be reached before this technique can be used, preventing the study of earlier stages of the tumor. The second way to use BT seeds in vivo is through the use of plesiotherapy, which involves placing them around or on the tumor externally. This technique is used in some cases of feline and canine cancer [19–21]. In the clinic, custom molds are used for nonmelanoma skin cancer treatment by Dr Algara Lopez’s team in Barcelona, Spain [27]. However, very few preclinical studies have used plesiotherapy. For example, in 1989, Jones et al. developed cap-bearing I-125 seeds for low-dose-rate irradiation of mice [28], and more recently, Jarosz-Biej et al. used a clinical brachytherapy setup with iridium-192 seeds for research on melanoma [29].

In the protocol described herein, seeds are placed directly on top of the tumor, allowing adaptation to tumor height and avoiding proximity to vital organs to preserve deep normal tissue. The technique is noninvasive and avoids possible inflammatory reactions linked with seed insertion. Additional seeds can be added for full coverage, and the radioactive sources can also be changed to study the dose rate, for example. The 3D-printed cases are inexpensive and simple to fabricate, and their design can be adapted to various tumor models and locations or experimental requirements. Both the seeds and the cases can be easily reused, further contributing to reducing the costs per experiment to a mere fraction of the price of a cabinet irradiator. Furthermore, this protocol offers convenient fractionation, avoiding the need for costly equipment and complex dose calculations, which can help in studies of BT dose (total dose or dose per fraction). Multiple tumors can be treated with various doses in the same animal by using several seed-containing cases and different exposure times. In comparison with EBRT, our technique requires a much simpler calculation of dose and is much more accurate because the seeds are placed in contact with the tumor, eliminating the need to include margins of error from misplacement of the animal and breathing motion [30]. However, one of the drawbacks of the plesiotherapy technique is the heterogeneity of the dose between the top of the tumor, closest to the skin, and the bottom of the tumor, deeper inside the tissues. The dose that can be delivered in one treatment is limited by possible skin toxicity and by the duration of the treatment (depending on seed activity and the desired target median dose), which needs to be applied to anesthetized animals. Another drawback of the plesiotherapy technique is that it is restricted to superficial/subcutaneous tumors and therefore is not suitable for most orthotopic cancer studies. However, heterotopic tumor models are technically simple, convenient and relevant to a wide variety of research topics and are still commonly used [18].

## Conclusions

This in vivo radiation protocol is a simple and economical way to irradiate tumor-bearing mice on demand, facilitating RT research in a variety of laboratory settings. The protocol can be adapted to various tumor shapes and sizes owing to the versatility of 3D printing and seed geometric allocations within the cartridge.

## Electronic supplementary material

Below is the link to the electronic supplementary material.

Supplementary Material 1

## Data Availability

The datasets used and/or analyzed during the current study are available from the corresponding author on reasonable request.

## Abbreviations

BT: Brachytherapy
CCAC: Canadian Council on Animal Care
DMEM: Dulbecco’s modified Eagle’s medium
EBRT: External beam radiotherapy
FBS: Fetal bovine serum
H&EL: Hematoxylin and eosin
I-125: Iodine 125
LINAC: Linear accelerator
NCCN: National Comprehensive Cancer Network
RPMI: Roswell Park Memorial Institute
RT: Radiotherapy
STS: Soft-tissue sarcoma
TNBC: Triple-negative breast cancer
